# Myoarchitectural disarray of hypertrophic cardiomyopathy begins pre‐birth

**DOI:** 10.1111/joa.13058

**Published:** 2019-07-26

**Authors:** Patricia Garcia‐Canadilla, Andrew C. Cook, Timothy J. Mohun, Onyedikachi Oji, Saskia Schlossarek, Lucie Carrier, William J. McKenna, James C. Moon, Gabriella Captur

**Affiliations:** ^1^ Institute of Cardiovascular Science University College London London UK; ^2^ The Francis Crick Institute London UK; ^3^ Cardiovascular Research Centre Institute of Experimental Pharmacology and Toxicology University Medical Center Hamburg‐Eppendorf Hamburg Germany; ^4^ DZHK (German Center for Cardiovascular Research), partner site Hamburg/Kiel/Lübeck Hamburg Germany; ^5^ The Cardiovascular Magnetic Resonance Imaging Unit Barts Heart Centre St Bartholomew's Hospital London UK

**Keywords:** cardiac embryology, developmental biology, hypertrophic cardiomyopathy, myocardial disarray

## Abstract

Myoarchitectural disarray – the multiscalar disorganisation of myocytes, is a recognised histopathological hallmark of adult human hypertrophic cardiomyopathy (HCM). It occurs before the establishment of left ventricular hypertrophy (LVH) but its early origins and evolution around the time of birth are unknown. Our aim is to investigate whether myoarchitectural abnormalities in HCM are present in the fetal heart. We used wild‐type, heterozygous and homozygous hearts (*n* = 56) from a *Mybpc3*‐targeted knock‐out HCM mouse model and imaged the 3D micro‐structure by high‐resolution episcopic microscopy. We developed a novel structure tensor approach to extract, display and quantify myocyte orientation and its local angular uniformity by helical angle, angle of intrusion and myoarchitectural disarray index, respectively, immediately before and after birth. In wild‐type, we demonstrate uniformity of orientation of cardiomyocytes with smooth transitions of helical angle transmurally both before and after birth but with traces of disarray at the septal insertion points of the right ventricle. In comparison, heterozygous mice free of LVH, and homozygous mice showed not only loss of the normal linear helical angulation transmural profiles observed in wild‐type but also fewer circumferentially arranged myocytes at birth. Heterozygous and homozygous showed more disarray with a wider distribution than in wild‐type before birth. In heterozygous mice, disarray was seen in the anterior, septal and inferior walls irrespective of stage, whereas in homozygous mice it extended to the whole LV circumference including the lateral wall. In conclusion, myoarchitectural disarray is detectable in the fetal heart of an HCM mouse model before the development of LVH.

## Introduction

Adverse clinical outcomes in patients with hypertrophic cardiomyopathy (HCM), such as cardiac arrhythmias, heart failure and sudden cardiac death (SCD), are thought to be related to changes in myocardial substrate including myoarchitectural disarray, fibrosis and small vessel disease. There is evidence that disarray precedes left ventricular hypertrophy (LVH) in at least some patients with HCM: in a postmortem histopathological study of subclinical HCM (before the development of LVH), the hearts of four related SCD victims with apparently normal mass and wall thickness, demonstrated widespread myoarchitectural disarray (McKenna et al. 1990) and a pathogenic HCM‐causing sarcomere gene mutation was later implicated. In another UK regional postmortem registry, nine hearts from athletes with SCD, which again showed normal wall thickness and mass, also had myoarchitectural disarray consistent with HCM (Finocchiaro et al. 2016). These data suggest that the subclinical HCM state with disarray but no LVH, may on occasion not be entirely benign and indeed can be associated with SCD. To pursue precision medicine for HCM and encouraged by recent advances in targeted molecular and genetic therapies for the disease (Ammirati et al. 2016; Prondzynski et al. 2018), we first need to understand the pathophysiological timings of disarray to ensure potential corrective strategies are started sufficiently early in patients with HCM.

Studying HCM in the search for the early origins of myoarchitectural disarray potentially offers insights into disease pathogenesis. Previous mouse work has suggested that sarcomeric protein mutations potentially influence cardiac development as they associate with morphological features identifiable *in utero* (Captur et al. 2016). Histopathologically, the adult mouse heart with LVH due to homozygous (HO) loss of cardiac myosin binding protein‐C (cMyBP‐C) is known to exhibit increased interstitial fibrosis, expansion of mononuclear cardiomyocytes size, myocyte mitochondria of reduced size, ultrastructural loss of lateral alignment of adjacent myofibrils and Z‐line misalignment, as well as myoarchitectural disarray (Harris et al. 2002; Korte et al. 2003; Carrier et al. 2004; Brickson et al. 2007; Luther et al. 2008). However, myoarchitectural disarray has not been quantitively assessed in any of these previous studies.

In the present study, we report a novel imaging framework quantitively to measure the orientation of myocytes and myoarchitectural disarray in fetal and postnatal murine hearts to find out whether myoarchitectural abnormalities in HCM are present before birth. We use a mouse model that genetically recapitulates human HCM together with high‐resolution episcopic microscopy (HREM) (Weninger et al. 2006) and a novel structure tensor approach to do this. HREM visualises the hearts and morphological features in three‐dimensions (3D) with a resolution of only few microns. The HCM mouse model is a *Mybpc3*‐targeted KO [knock‐out (KO)] (Carrier et al. 2004) based upon the targeted deletion of exons 1 and 2 from the endogenous *Mybpc3* gene, containing the transcription initiation site with consequent protein ablation.

## Materials and methods

### Animals

All mice (*Mus musculus*) were handled in accordance with the Guide for the Care and Use of Laboratory Animals published by the U.S. National Institutes of Health and with the approval of the University Medical Centre Hamburg‐Eppendorf Ethical Review Panel. Procedures were in accordance with the German Law for the Protection of Animals and accepted by the Ministry of Science and Public Health of the City State of Hamburg, Germany (Nr. ORG 696). Both KO and wild‐type mice were maintained on a C57Bl/6J background at the University Medical Centre Hamburg‐Eppendorf as previously described (Captur et al. 2016). For approximate embryo staging, detection of a vaginal plug was taken as gestation day 0.5. Pregnant females were euthanised by CO_2_ inhalation and sacrificed to permit embryo harvesting.

Mice were staged according to gestational age [embryonic days (E)] such that E18.5 is immediately before birth and P0 (postnatal day 0), the neonatal mouse. A total of 56 embryo hearts, of which 33 at E18.5 (8 wild‐type, 7 heterozygous, 18 homozygous) and 23 at P0 (4 wild‐type, 9 heterozygous, 10 homozygous) were isolated as previously described (Mohun & Weninger, 2012). Hearts were dehydrated, infiltrated with methacrylate resin and used for HREM analysis (Weninger et al. 2006). Each HREM image stack comprised up to 1300 short axis images, produced by successive removal of 3‐μm sections. The mean ± standard deviation pixel size was 30.03 ± 0.27 μm^2^ and voxel size 164.57 ± 0.14 μm^3^: for comparison, a healthy adult mice sarcomere is *ca*. 1.6–2.2 μm in length at rest (Bub et al. 2010) and each myocyte measures *ca*. 100 μm long by 15–20 μm wide (Olivetti et al. 1996; Göktepe et al. 2010).

### Image processing and analysis of murine data

**Figure 1:**
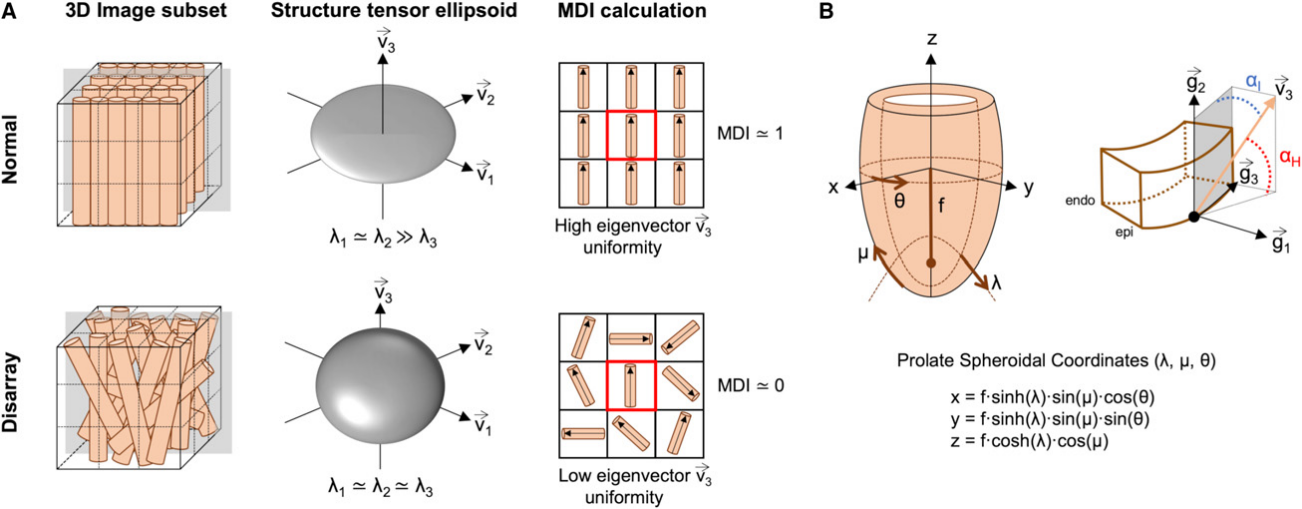
Normal myoarchitectural organisation and definition of local coordinates system and eigenvectors. In health, human cardiomyocytes are aggregated in bundles. Their helical orientation changes with progression through the thickness of the left ventricular (LV) wall with: left‐handed (negative angulation) longitudinally arranged cardiomyocytes in the epicardium; circumferentially arranged (0**°** angulation) cardiomyocytes in the mid‐wall; and right‐handed (positive angulation) longitudinally arranged cardiomyocytes in the endocardium (MacIver et al. 2018; Stephenson et al. 2018). We have recently reported how this gradual change in helical angulation (HA) is established early in fetal life (Garcia‐Canadilla et al. 2018). Cardiomyocytes also show a transmural orientation which is quantified by the transverse (TA) or intrusion angle. (a) Schematic representation of the region of interest (ROI) defined to calculate the structure tensor in a given voxel, formed by the voxel itself (in the centre of the cube – red square in left panel) and their eight nearest voxels. In this drawing, each cylinder represents a single cardiomyocyte. The middle panel illustrates the eigenvector system (***v***_***i***_, i = 1…3) and their ellipsoids obtained with structure tensor analysis in healthy (normal) vs. hypertrophic cardiomyopathy (disarray) showing high anisotropy represented by a flat ellipsoid (organised myocardium) vs. low anisotropy represented by a more spherical ellipsoid (disorganised myocardium). The right panel illustrates the calculation of the myoarchitectural disarray index (MDI) in the same ROI used to calculate the structure tensor. In this drawing, we have plotted the tertiary eigenvectors in the 2D plane indicated in grey in the right panel. MDI in a given voxel quantifies the collinearity or angular uniformity between the tertiary eigenvectors of the voxel itself (red square) and the tertiary eigenvectors of their nearest neighbours. In a normal/healthy cardiac tissue, all the eigenvectors have similar orientations, which means that they are highly collinear and therefore MDI is close to 1. However, when disarray is present, all the eigenvectors within the ROI have very different orientations, which means that the collinearity between all eigenvectors is very low and therefore MDI is close to 0. (b) Scheme defining local prolate spheroidal coordinates of the LV, with transmural (λ), longitudinal (μ) and circumferential (θ) axis and the helical (HA) and intrusion (IA) angles used to describe myocyte orientation. HA, denoted α_H_, is defined as the angle between the local short‐axis or circumferential plane and the tertiary eigenvector ***v***_3_. IA, denoted α_I_, is the angle between the local epicardial tangential plane and the tertiary eigenvector ***v***_3_. The vectors ***g***_1_, ***g***_2_ and ***g***_3_ form the prolate spheroidal coordinates basis and are calculated as: $\vec{g_{1}}=f\frac{\partial \chi }{\partial \lambda }$, $\vec{g_{2}}=f\frac{\partial \chi }{\partial \mu }$ and $\vec{g_{3}}=f\frac{\partial \chi }{\partial \theta }$, where χ = (*x*,* y*,* z*) are the Cartesian coordinates, and *f* is the semi‐foci distance.

Acquired HREM datasets were first isotropically resampled to obtain the same pixel size in the three directions, *x*,* y* and *z*, followed by automatic segmentation in fiji (Schindelin et al. 2012). 3D volume‐rendered reconstructions were performed in 3dslicer (Fedorov et al. 2012). The orientation of cardiomyocytes was assessed by computation of their helical and intrusion angles by means of an in‐house structure tensor method implemented in matlab **®** (The MathWorks Inc., Natick, MA, USA, R2018a) (Baličević et al. 2015; Garcia‐Canadilla et al. 2018). The structure tensor method is an image analysis method which derives a tensor from the distribution of image gradient directions within the neighbourhood of a voxel. Details on the calculation of myocyte orientation are provided in Supporting Information Appendix S1. The helical angle was calculated as the angle between the tertiary eigenvector ***v***_3_ (the vector pointing in the longitudinal direction of the cardiomyocytes), and the local circumferential plane defined by the local transmural (*g*_1_) and circumferential (*g*_3_) directions (Fig. 1a). Angle of intrusion was calculated as the angle between the tertiary eigenvector ***v***_3_ and the local epicardial tangential plane, defined by the local longitudinal (*g*_2_) and circumferential (*g*_1_) directions (Fig. 1a). Distributions of helical and intrusion angles at apical, midventricular and basal levels as well as in each LV segment recapitulating the American Heart Association 17‐segment cardiac model (apical segment 17 not analysed) were obtained. Circumferential cardiomyocytes will have helical angle close to 0°. Phase wrapping of the helical angle around +90°, as tends to occur particularly at the papillary muscle insertion points, was avoided by expanding the dynamic range (from +150° to −90°). Transmural profiles of helical and intrusion angles from endo‐ to epicardium were obtained for each LV segment. Next, a linear regression fitting, *y = *β_1_ *·x *+* *β_0_, was applied to transmural helical angle profiles to characterise their linearity. *R* ^2^ coefficient and gradient over the myocardial wall in °/μm (β_1_) were computed.

To quantify myoarchitectural disarray we computed the myoarchitectural disarray index (MDI) similar to the manner used in diffusion tensor magnetic resonance imaging (DT‐MRI) (Wu et al. 2004; Wang et al. 2008; Giannakidis et al. 2012). This index, which is equivalent to the intervoxel coherence index described by Giannakidis et al. (2012), quantifies, for each image voxel, the uniformity of the myocyte longitudinal direction (represented by the tertiary eigenvector ***v***_3_, Fig. 1b) within a neighbourhood that consists of the voxel itself and its nearest neighbours. In the current study, the computations were performed using an ROI of 9 × 9 × 9 voxels, which represents a volume of 49.3 × 49.3 × 49.3 μm^3^. Details on the calculation of the MDI are provided in Supporting Information Appendix S2. MDI takes values from 0 to 1. A large value of MDI at a given voxel indicates that the disarray is insignificant in the neighbourhood of this voxel (all the myocytes within the neighbourhood have similar orientations), whereas smaller values of MDI denote a great loss of myocyte organisation or, in other words, high degree of myoarchitectural disarray (all the myocytes within the neighbourhood have very different orientations; Fig. 1b).

To ensure better LV coverage and resolution to pick up even small regions of disarray, we analysed MDI and LV wall thickness using a 45 myocardial segment model (Supporting Information Fig. S1). For wall thickness, this consisted of nine radial caliper spoke measurements at each of five levels from LV base to apex. For MDI, each of the 45 segments was further subdivided into four equal layers: inner, mid‐inner, mid‐outer and outer, starting at the endocardium. Mean MDI per layer, per segment was then computed.

### 3D vector representation and tracking of myocytes

3D plots of the longitudinal direction of the myocytes, represented by the tertiary eigenvector ***v***_3_, in mid LV short‐axis slices were performed in paraview (Ahrens et al. 2005) using a vector Glyph filter. 3D vectors were displayed after applying a tube filter and color‐coded by *z*‐component of the tertiary eigenvector ***v***_3_. Additionally, a tractography algorithm was also applied in the whole heart to track through the tertiary eigenvector ***v***_3_, using a 2nd order Runge‐Kutta method. In this way, tertiary eigenvectors were concatenated voxel‐to‐voxel to form tracks. These tracks were considered representative of the local geometrical arrangement of the cardiomyocytes. Tracks were displayed after applying a tube filter in the whole heart (see Supporting Information Videos S1–S3).

### Statistical analysis

Statistical analysis was performed in SPSS® (IBM Corp. Released 2016. IBM SPSS Statistics for Macintosh, Version 24.0; IBM Corp., Armonk, NY, USA). Illustrations reporting bullseye plots were constructed in matlab® (The MathWorks Inc., R2018a). Descriptive data are expressed as mean ± standard deviation except where stated otherwise. Distribution of data was assessed on histograms and using the Shapiro–Wilk test. Normally distributed continuous variables pertaining to wild‐type, heterozygous and homozygous mice were compared using analysis of variance (anova) with Tukey's post‐hoc test. Non‐normally distributed continuous variables were compared using the Kruskal–Wallis test. Pearson's correlation coefficient was used to explore the relationship of MDI to LV wall thickness normalised to LV base‐to‐apical length. Differences between mutant and wild‐type groups and between developmental stages of the distributions of HA and TA were tested using the two‐sample Kolmogorov–Smirnov test. To compare non‐parametric MDI histograms between developmental stages, and between mutant and wild‐type mouse populations, we used fitted generalised additive models implemented with a Gauss–Seidel backfitting algorithm (object ‘gam’ in package ‘mgcv’). Models were constructed for the analysis of response MDI to predictor terms *A* and *X*, where *A* was the fixed factor (mouse group or developmental stage) and covariate *X* a numeric vector of 101 different elements from 0 to 1. In all statistical tests, a two‐sided *P*‐value < 0.05 was considered significant.

## Results

### Normal myoarchitecture in the fetal and postnatal mouse

**Figure 2:**
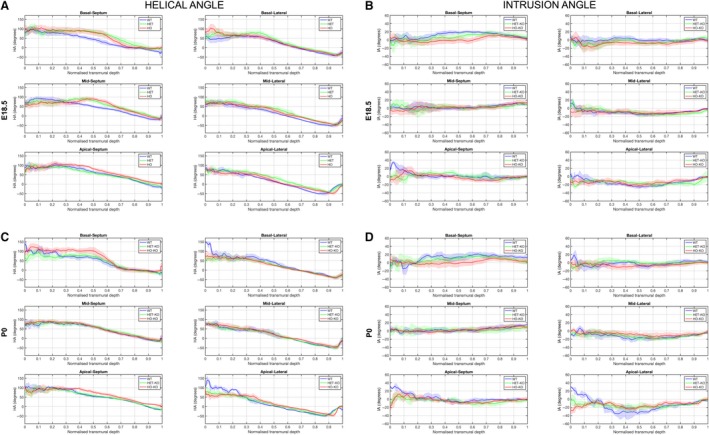
Transmural LV change in helical (HA) and intrusion (IA) angle. Left ventricular (LV) transmural profiles of helical angle (HA) across septal (segments 2, 3 for the base; 8, 9 for middle; segment 14 for the apex) and lateral walls (segments 5, 6 for the base; 11, 12 for middle; segment 16 for the apex) for wild‐type (WT), heterozygous (HET) and homozygous (HO) knock‐out mice at (a) embryonic day (E) 18.5 and (c) post‐natal day (P) 0. LV transmural profile of intrusion angle (IA) across septal and lateral walls (same segments than HA) for WT, HET and HO knock‐out mice at (b) E18.5 and (d) P0. Solid lines: group means; Ribbons: ± standard deviation.

**Figure 3:**
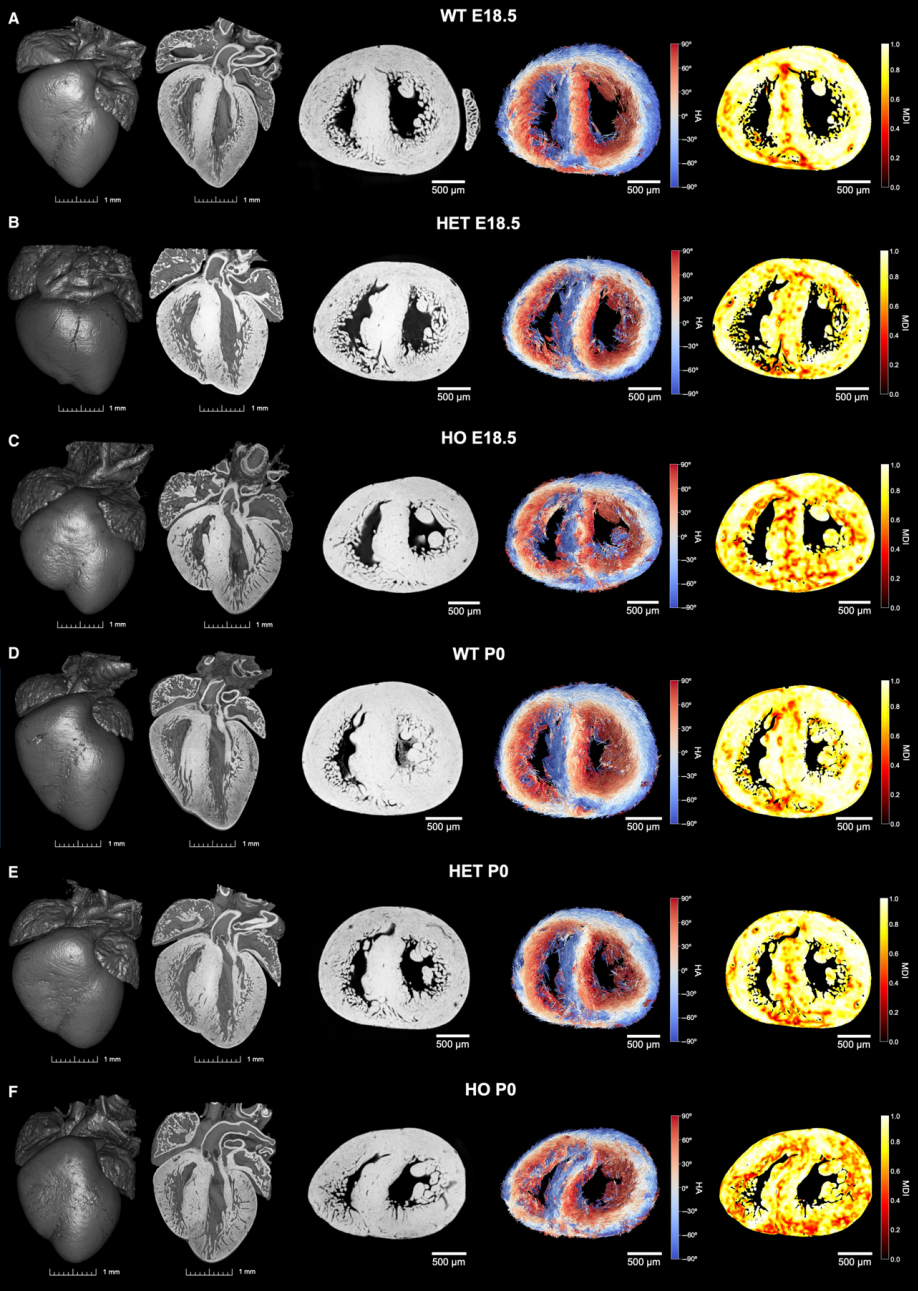
Volumetric representation of the developing murine heart and matching fibre tracking and myoarchitectural disarray index (MDI) colour maps for the mid LV. Whole‐heart volume‐rendered three‐dimensional (3D) high‐resolution episcopic microscopy reconstruction in (a,d) WT, (b,e) HET and (c,f) HO knock‐out mice at two stages: E18.5 (a–c) and P0 (d–f). LV hypertrophy is apparent in both HO and HET. Black/white inverted original episcopic microscopy images, myocyte tracking and MDI colour maps for a single representative mid LV slice are shown on the right. Other abbreviations as in Fig. 2.

**Figure 4:**
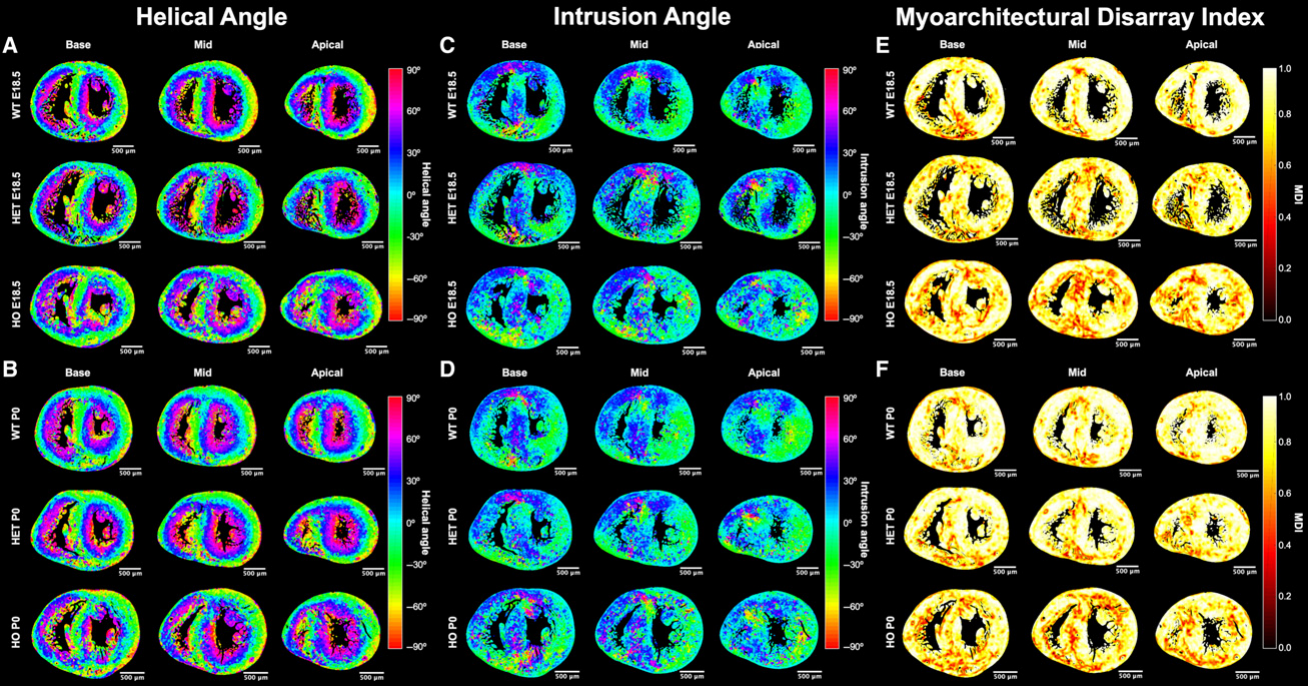
Quantification of myocyte orientation (via helical and intrusion angles) and myoarchitectural disarray (via MDI). Left panel: local HA at three levels from LV base to apex at (a) E18.5 and (b) P0 in WT (top), HET (middle) and HO (bottom). Middle panel: local IA at three levels from LV base to apex at (c) E18.5 and (d) P0 in WT (top), HET (middle) and HO (bottom). Right panel: MDI at three levels from LV base to apex at (e) E18.5 and (f) P0 in WT (top), HET (middle) and HO (bottom). High MDI (near 1, yellow/white spectrum extreme) implies insignificant disarray, whereas low MDI (burgundy/black spectrum extreme) denotes disarray. Other abbreviations as in Fig. 2.

**Figure 5:**
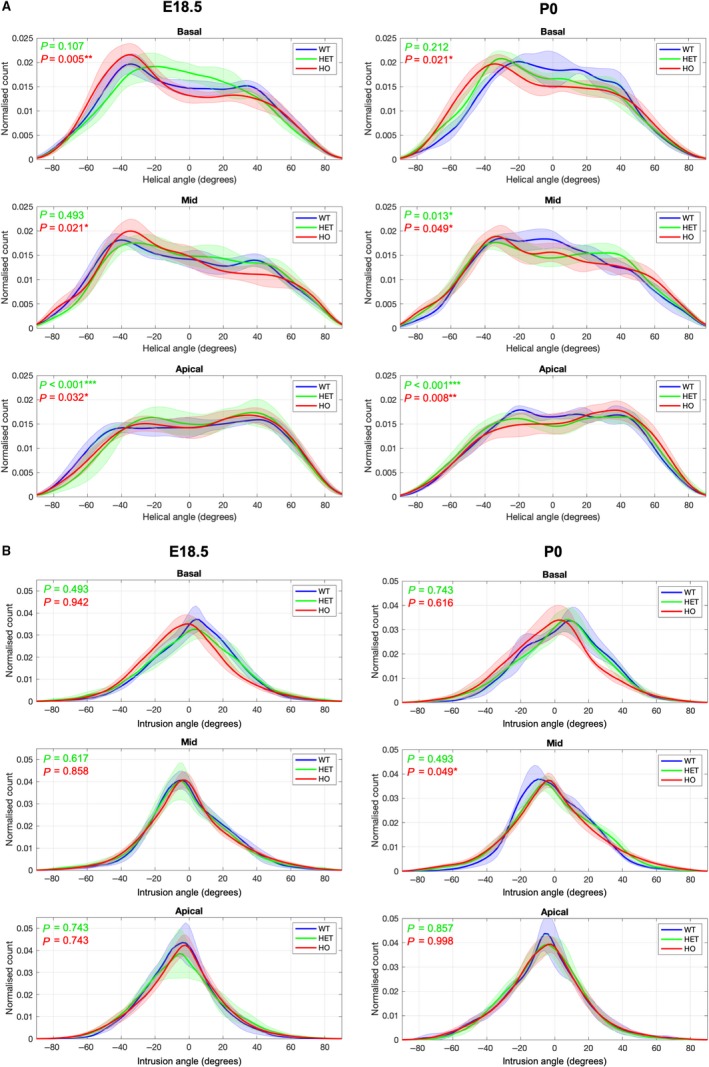
Averaged distribution of helical and intrusion angles in the LV. Histograms of (a) HA and (b) IA showing mean values across the basal, midventricular and apical LV slices of WT, HET and HO knock‐out mouse hearts at E18.5 (left) and P0 (right). Solid lines: group means. Ribbons: ± standard deviation. *Significantly different from wild‐type (WT) mice at same stage, *P *<* *0.05; **Significantly different from WT same stage, *P *<* *0.01; ***Significantly different from WT same stage, *P *<* *0.001. Other abbreviations as in Fig. 2.

Wild‐type myocardium demonstrated an orderly ‘normal’ architecture, both pre‐ and postnatally, showing a smooth and gradual change of the helical angle of cardiomyocytes along the LV wall depth from +90**°** in the endocardium to −50**°** in the epicardium or right‐sided endocardium (Fig. 2a,c; Supporting Information Fig. S2). We also observed that circumferential bundles of myocytes predominated in the LV midwall in wild‐type as expected (white midwall 3D vector plots in Fig. 3a,d and turquoise midwall helical angle signal in Fig. 4a,b). Linearity values for helical angle transmural profiles (*R* ^2^) were significantly higher in wild‐type than in mutants, indicating a smooth and linear change in the helical angle transmurally in wild‐type (basal anteroseptum wild‐type vs. heterozygous mice at E18.5 and P0: 0.83 ± 0.25 vs. 0.52 ± 0.22 and 0.78 ± 0.25 vs. 0.52 ± 0.24, respectively, *P *<* *0.05 for E18.5, Table 1) and they tended to be higher (albeit not achieving statistical significance) in wild‐type P0 than wild‐type E18.5 (mean *R* ^2^ 0.83 and 0.80, respectively; Table 1). There is a significant developmental increase in the percentage of circumferentially arranged myocytes (−10**°** < helical angle < 10**°**) observed at the mid LV myocardium (wild‐type E18.5 vs. wild‐type P0: 15.5 ± 1.9% vs. 19.7 ± 1.8%, *P* = 0.014; Table 2; Fig. 5a). Figure 5a shows the distribution of helical angles in basal, midventricular and apical slices of the wild‐type before and after birth. Detailed analyses on the different 16 LV sections can be found in Supporting Information Fig. S4. We observed considerable local variability of the helical angle when comparing the different LV segments (Fig. S4). For example, myocytes are aligned more longitudinally with respect to the equatorial plane in the midventricular and basal inferior wall than in the anterior wall.

**Table 1:** HREM summary data

	Wild‐type		Heterozygous		Homozygous	
	E18.5	P0	E18.5	P0	E18.5	P0
No. of mice	8	4	7	9	18	10
LV max. septal WT^a^	0.495 ± 0.059	0.598 ± 0.133	0.507 ± 0.077	0.568 ± 0.040	0.528 ± 0.044	0.566 ± 0.053
LV length (mm)	2.33 ± 0.11	2.54 ± 0.06	2.25 ± 0.15	2.38 ± 0.12	2.18 ± 0.12***	2.11 ± 0.06* ^,^ †
Slope (β_1_) of the linear fitting of helical angle transmural profile (^o^/μm)						
Basal Anterior	−0.20 ± 0.05	−0.23 ± 0.04	−0.19 ± 0.03	−0.16 ± 0.04***	−0.20 ± 0.07	−0.23 ± 0.04††
Basanteroseptal	−0.31 ± 0.09	−0.31 ± 0.10	−0.21 ± 0.08	−0.21 ± 0.07	−0.27 ± 0.17	−0.37 ± 0.05††
Inferoseptal	−0.27 ± 0.07	−0.29 ± 0.05	−0.26 ± 0.03	−0.25 ± 0.08	−0.31 ± 0.14	−0.36 ± 0.05†††
Inferior	−0.18 ± 0.09	−0.23 ± 0.04	−0.25 ± 0.09	−0.23 ± 0.08	−0.21 ± 0.07	−0.24 ± 0.05
Inferolateral	−0.21 ± 0.03	−0.22 ± 0.07	−0.23 ± 0.05	−0.21 ± 0.06	−0.24 ± 0.06	−0.22 ± 0.02
Anterolateral	−0.20 ± 0.04	−0.24 ± 0.04	−0.19 ± 0.04	−0.20 ± 0.05	−0.22 ± 0.05	−0.22 ± 0.03
Mid Anterior	−0.15 ± 0.03	−0.11 ± 0.02	−0.14 ± 0.04	−0.16 ± 0.02***	−0.14 ± 0.03	−0.15 ± 0.02***
Anteroseptal	−0.22 ± 0.04	−0.15 ± 0.04	−0.12 ± 0.06***	−0.19 ± 0.05	−0.12 ± 0.08**	−0.23 ± 0.05***
Inferoseptal	−0.22 ± 0.04	−0.18 ± 0.03	−0.15 ± 0.06***	−0.16 ± 0.03	−0.14 ± 0.08***	−0.19 ± 0.05
Inferior	−0.14 ± 0.02	−0.13 ± 0.01	−0.14 ± 0.02	−0.13 ± 0.02	−0.14 ± 0.03	−0.15 ± 0.02
Inferolateral	−0.19 ± 0.01	−0.16 ± 0.02	−0.20 ± 0.05	−0.18 ± 0.02	−0.17 ± 0.03	−0.19 ± 0.03
Anterolateral	−0.20 ± 0.02	−0.15 ± 0.02	−0.21 ± 0.03	−0.19 ± 0.01***	−0.18 ± 0.03†††	−0.19 ± 0.03***
Apical anterior	−0.19 ± 0.03	−0.20 ± 0.03	−0.17 ± 0.02	−0.19 ± 0.04	−0.16 ± 0.03	−0.20 ± 0.04
Septal	−0.25 ± 0.04	−0.20 ± 0.03	−0.25 ± 0.03	−0.23 ± 0.05	−0.18 ± 0.05*** ^,^ ††	−0.20 ± 0.06
Inferior	−0.19 ± 0.03	−0.17 ± 0.02	−0.21 ± 0.01	−0.18 ± 0.03	−0.16 ± 0.03*** ^,^ †	−0.17 ± 0.04
Lateral	−0.20 ± 0.03	−0.18 ± 0.01	−0.23 ± 0.03	−0.19 ± 0.02***	−0.18 ± 0.03††	−0.18 ± 0.02**
Linearity (*R* ^2^) of the linear fitting of helical angle transmural profile (Unitless)						
Basal anterior	0.69 ± 0.20	0.91 ± 0.09	0.67 ± 0.14	0.56 ± 0.16**	0.71 ± 0.26	0.84 ± 0.08††
Anteroseptal	0.83 ± 0.26	0.78 ± 0.25	0.52 ± 0.22***	0.52 ± 0.24	0.60 ± 0.28***	0.73 ± 0.12†††
Inferoseptal	0.76 ± 0.22	0.81 ± 0.05	0.64 ± 0.10***	0.60 ± 0.23	0.64 ± 0.29	0.74 ± 0.09***
Inferior	0.67 ± 0.31	0.73 ± 0.11	0.69 ± 0.28	0.75 ± 0.11	0.55 ± 0.20	0.76 ± 0.05
Inferolateral	0.80 ± 0.12	0.76 ± 0.14	0.72 ± 0.11	0.81 ± 0.12	0.76 ± 0.14	0.87 ± 0.06
Anterolateral	0.70 ± 0.18	0.91 ± 0.08	0.71 ± 0.13	0.76 ± 0.12	0.82 ± 0.16	0.89 ± 0.05††
Mid anterior	0.81 ± 0.07	0.77 ± 0.11	0.65 ± 0.18***	0.85 ± 0.06	0.65 ± 0.21***	0.80 ± 0.09
Anteroseptal	0.82 ± 0.12	0.66 ± 0.13	0.46 ± 0.30**	0.73 ± 0.18	0.38 ± 0.23**	0.86 ± 0.08***
Inferoseptal	0.86 ± 0.15	0.84 ± 0.15	0.53 ± 0.28**	0.76 ± 0.13	0.49 ± 0.28**	0.76 ± 0.11
Inferior	0.81 ± 0.08	0.90 ± 0.02	0.71 ± 0.12	0.81 ± 0.09	0.80 ± 0.12†††	0.85 ± 0.08
Inferolateral	0.87 ± 0.06	0.86 ± 0.07	0.84 ± 0.09	0.87 ± 0.06	0.84 ± 0.09	0.90 ± 0.03
Anterolateral	0.91 ± 0.04	0.93 ± 0.03	0.87 ± 0.07	0.92 ± 0.03	0.86 ± 0.10	0.90 ± 0.06
Apical anterior	0.80 ± 0.14	0.84 ± 0.11	0.71 ± 0.08***	0.83 ± 0.17	0.75 ± 0.12	0.81 ± 0.13
Septal	0.80 ± 0.13	0.86 ± 0.09	0.85 ± 0.07	0.80 ± 0.13	0.65 ± 0.18††	0.72 ± 0.25
Inferior	0.82 ± 0.11	0.95 ± 0.03	0.82 ± 0.10	0.92 ± 0.04	0.78 ± 0.14	0.81 ± 0.10*** ^,^ †††
Lateral	0.83 ± 0.07	0.84 ± 0.03	0.87 ± 0.04	0.86 ± 0.03	0.86 ± 0.05	0.83 ± 0.03
Myoarchitectural disarray index (MDI) (Unitless)						
Basal anterior	0.78 ± 0.04	0.78 ± 0.07	0.71 ± 0.04***	0.76 ± 0.05	0.73 ± 0.04	0.77 ± 0.06
Anteroseptal	0.82 ± 0.02	0.83 ± 0.02	0.70 ± 0.20**	0.81 ± 0.01	0.74 ± 0.03* ^,^ †††	0.79 ± 0.04
Inferoseptal	0.74 ± 0.02	0.74 ± 0.04	0.68 ± 0.13	0.74 ± 0.04	0.72 ± 0.05	0.72 ± 0.04
Inferior	0.72 ± 0.05	0.74 ± 0.04	0.73 ± 0.08	0.76 ± 0.04	0.70 ± 0.04	0.70 ± 0.04†††
Inferolateral	0.78 ± 0.05	0.76 ± 0.03	0.78 ± 0.05	0.80 ± 0.04***	0.77 ± 0.03	0.77 ± 0.04
Anterolateral	0.80 ± 0.03	0.84 ± 0.03	0.76 ± 0.02***	0.80 ± 0.03	0.78 ± 0.03	0.80 ± 0.03***
Mid anterior	0.80 ± 0.03	0.81 ± 0.02	0.78 ± 0.03	0.79 ± 0.04	0.74 ± 0.02** ^,^ †††	0.75 ± 0.03***
Anteroseptal	0.81 ± 0.02	0.84 ± 0.02	0.75 ± 0.07***	0.81 ± 0.04	0.74 ± 0.03*	0.78 ± 0.02***
Inferoseptal	0.79 ± 0.03	0.79 ± 0.02	0.69 ± 0.11***	0.79 ± 0.03	0.69 ± 0.04*	0.72 ± 0.03** ^,^ †
Inferior	0.85 ± 0.03	0.87 ± 0.03	0.82 ± 0.04	0.86 ± 0.03	0.76 ± 0.05** ^,^ †††	0.77 ± 0.03** ^,^ †
Inferolateral	0.87 ± 0.03	0.84 ± 0.01	0.86 ± 0.03	0.86 ± 0.02	0.78 ± 0.05** ^,^ ††	0.75 ± 0.04** ^,^ †
Anterolateral	0.87 ± 0.03	0.84 ± 0.02	0.86 ± 0.02	0.82 ± 0.05	0.78 ± 0.03* ^,^ †	0.74 ± 0.03** ^,^ ††
Apical Anterior	0.81 ± 0.03	0.83 ± 0.03	0.80 ± 0.03	0.79 ± 0.04	0.70 ± 0.03* ^,^ †	0.71 ± 0.04** ^,^ †
Septal	0.81 ± 0.03	0.86 ± 0.03	0.76 ± 0.05	0.83 ± 0.03	0.78 ± 0.02	0.79 ± 0.03*** ^,^ †††
Inferior	0.81 ± 0.03	0.82 ± 0.04	0.79 ± 0.02	0.82 ± 0.03	0.78 ± 0.03***	0.79 ± 0.03
Lateral	0.82 ± 0.05	0.81 ± 0.02	0.80 ± 0.02	0.79 ± 0.04	0.73 ± 0.03** ^,^ †	0.70 ± 0.04** ^,^ ††

All values are expressed as mean ± standard deviation.

E, embryonic day; HREM, high‐resolution episcopic microscopy; LV, left ventricle; WT, wall thickness.

^1^Measurements of maximal LV wall thickness at the base (Levels –1 of 5). Complete wild‐type data is presented in Table S1.

*Significantly different from wild‐type mouse hearts at same stage, *P *<* *0.001.

**Significantly different from wild‐type same stage, *P *<* *0.01.

***Significantly different from wild‐type same stage, *P *<* *0.05.

^†^Significantly different from HET same stage, *P *<* *0.001.

^††^Significantly different from HET same stage, *P *<* *0.01.

^†††^Significantly different from HET same stage, *P *<* *0.05.

**Table 2:** Percentage of the circumferentially arranged myocytes at the three different heights of the LV: apical, mid and basal.

	Wildtype		Heterozygous		Homozygous	
	E18.5	P0	E18.5	P0	E18.5	P0
No. of mice	8	4	7	9	18	10
Percentage of circumferentially arranged myocytes (%)						
Apical	15.7 ± 2.1	18.3 ± 0.6	16.6 ± 2.7	16.3 ± 1.7***	15.8 ± 2.1	16.6 ± 2.2***
Mid	15.5 ± 1.9	19.7 ± 1.8†††	16.4 ± 2.1	16.1 ± 2.0***	16.1 ± 2.1	16.9 ± 2.1***
Basal	16.3 ± 1.7	20.5 ± 3.8	19.6 ± 2.1***	16.6 ± 1.5	14.6 ± 1.6***	16.6 ± 1.5***

All values are expressed as mean ± standard deviation unless otherwise stated.

E, embryonic day; HREM, high‐resolution episcopic microscopy; LV, left ventricle; WT, wall thickness.

***Significantly different from wild‐type same stage, *P *<* *0.05.

^†††^Significantly different from wild‐type E18.5, *P *<* *0.05.

**Figure 6:**
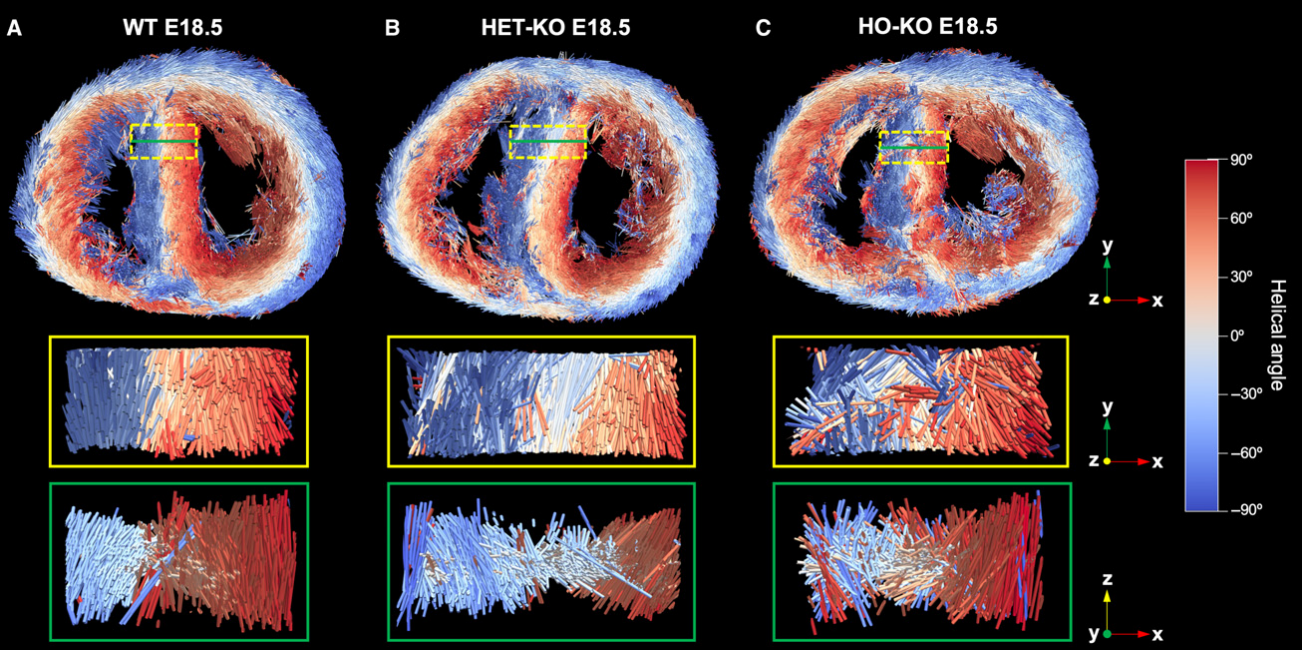
Myocyte tracking in fetal murine hearts. 3D representation of the principal vector (tertiary eigenvector ***v***_3_) pointing in the long axis of cardiomyocytes for a single representative midventricular LV slice (top) in (a) WT, (b) HET and (c) HO knock‐out mice together with a zoom in the inferior septum (dashed yellow rectangle) in *y*–*x* or parallel (middle) and *z*–*x* or perpendicular (bottom) views, which illustrates local myoarchitectural disarray in HET and HO compared with WT mice. Other abbreviations as in Figs 2 and 3.

Regarding the angle of intrusion, Fig. 2b,d shows its transmural course in basal, midventricular and apical slices of the LV lateral and septal walls. Detailed analyses on the different 16 LV sections can be found in Supporting Information Fig. S3. We noted a change from prevalence of positive angles of intrusion at the base to negative angles at the apex in wild‐type, as have been previously described in the murine adult heart (Schmitt et al. 2009). The distributions of angle of intrusion (Fig. 5b; Supporting Information Fig. S5) show that there is a high proportion of cardiomyocytes that deviate markedly from the tangential plane, with mean positive intruding angles at the base and mean negative intruding angles at midventricular and apical slices (Fig. 5b). For example, the proportion of myocytes with intruding angles higher than ± 20**°** in the midventricular level is about 36 and 37% at E18.5 and P0, respectively. The septum shows the widest distribution of angle of intrusion, indicating a higher proportion of myocytes with large intruding angles (Figs 6a and S5).

**Figure 7:**
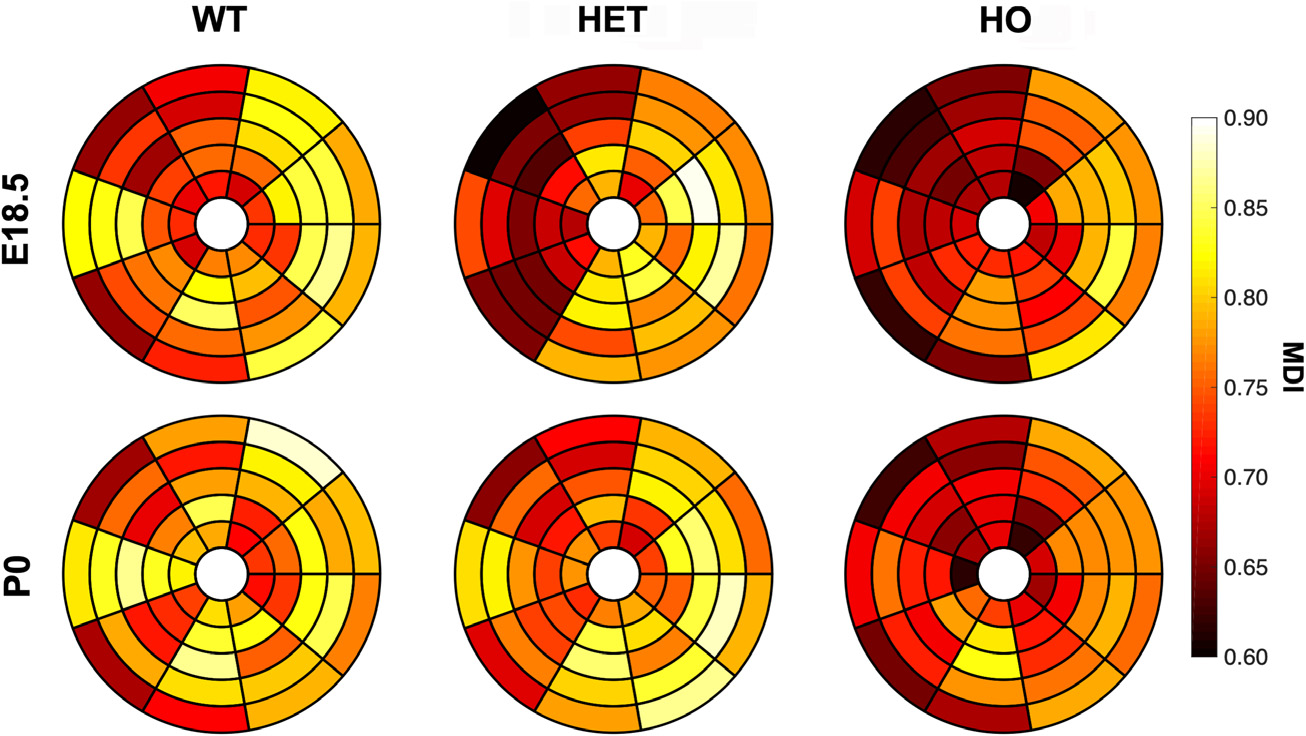
Bull's eye plots quantifying myoarchitectural disarray in developing murine HCM hearts. The 45‐segment bullseye plots of MDI at five levels from LV base to apex (nine segments per level) for WT, HET and HO mice at E18.5 and P0. Small MDI values (deep burgundy) denote a greater degree of myoarchitectural disarray. Other abbreviations as in Figs 2 and 3.

Wildtype myocardium showed uniformity of cardiomyocyte orientation within the region of interest, as MDI was generally > 0.70 irrespective of stage (mean MDI 0.81 both stages), indicating normal LV myocardial organisation and trivial disarray burden, both *in utero* and immediately after birth. In wild‐type, we observed small foci of low myocyte organization, i.e. disarray (voxels of MDI < 0.5 appearing as red/burgundy zones; Figs 3a,d, 4e,f and 7) at the superior and inferior right ventricular (RV) insertion points, and to a lesser extent around the trabeculated myocardium and blood vessels, and midwall along the septum.

### HCM mutants exhibit myoarchitectural disarray before and after birth

Heterozygous mutants were indistinguishable from wild‐type on the basis of maximal wall thickness at both E18.5 and P0 (no significant differences in wall thickness, see Supporting Information Table S1). At E18.5, transmural profiles of helical angle in mutants were less linear than wild‐type in the basal‐to‐mid anterior, septal and inferior segments. At P0, differences persisted at the base for heterozygous, and at the base and apex for homozygous mice (Table 1; Figs 2 and S2). Compared with wild‐type, the number of circumferentially arranged cardiomyocytes was significantly lower in both heterozygous and homozygous mice at birth (Table 2; Fig. 5a).

The overall distribution of angles of intrusion within the whole LV myocardial slices was not significantly different from the wild‐type (Fig. 5b). However, intrusion angles were significantly larger in the basal anterolateral wall and in the midventricular inferior, inferolateral and anterolateral walls of fetal mutant hearts, as demonstrated by the wider distributions of angles of intrusion (Fig. S5a), which correlate with the areas within the LV myocardium with high heterogeneity in the intrusion angle colour maps (Fig. 4c,d). Moreover, results of the 3D vector plots, which represent the local long axis of the myocytes, also show the larger values of angle of intrusion within the midventricular anteroseptal wall in the fetal mutant hearts (Fig. 6). At P0, we observed significant larger values of intrusion angle at the basal anteroseptal and anterolateral walls, and at the midventricular lateral, anteroseptal and inferior walls of homozygous mice, whereas in heterozygous mice this difference was only significant in the midventricular inferior wall (Fig. S5b).

**Figure 8:**
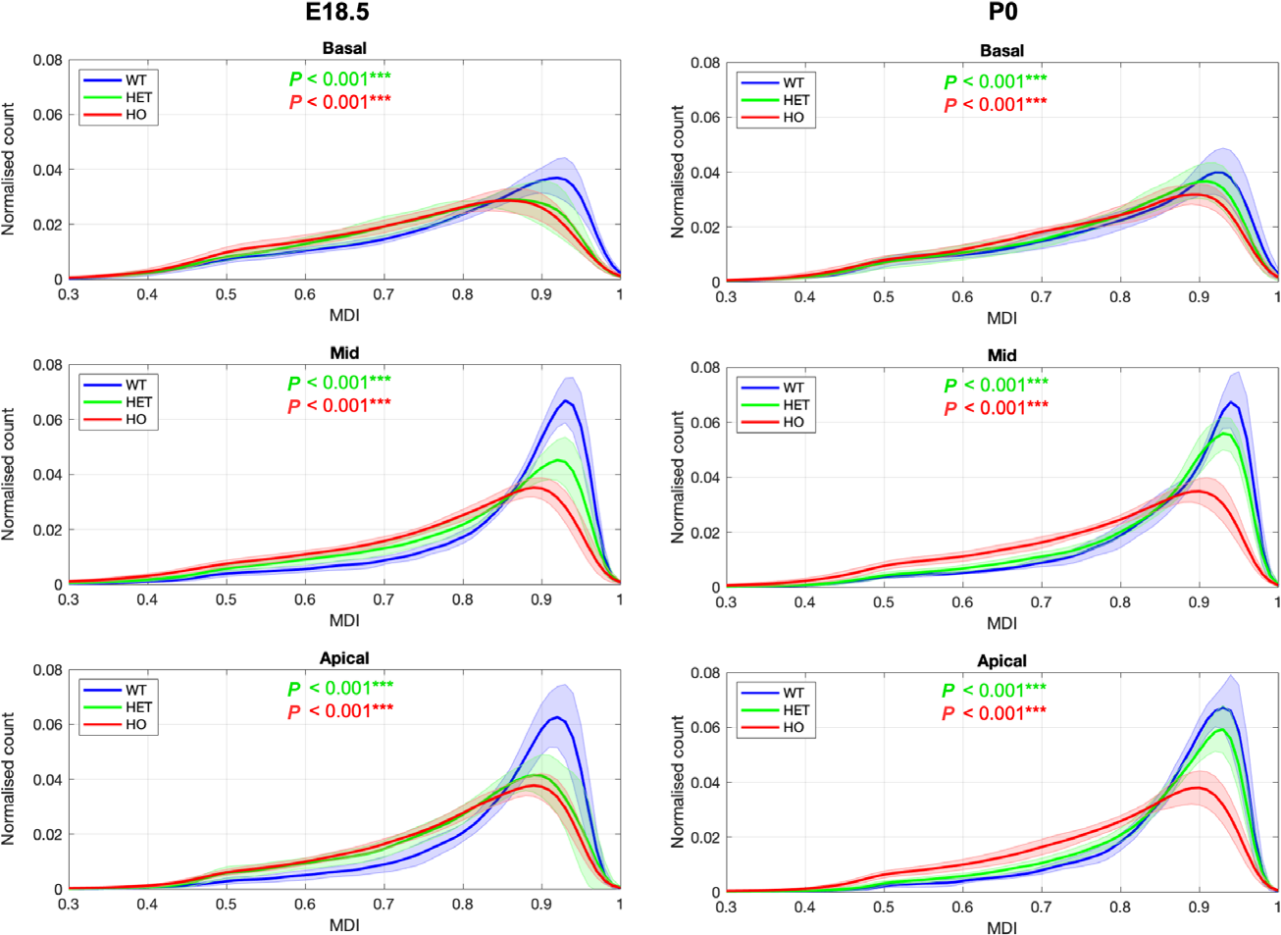
Averaged distribution of MDI in the LV. Histograms of MDI showing mean values across the basal, midventricular and apical LV slices of WT, HET and HO knock‐out mouse hearts at E18.5 (left) and P0 (right). Solid lines: group means. Ribbons: ± standard deviation. *Significantly different from WT same stage, *P *<* *0.05; **Significantly different from WT same stage, *P *<* *0.01; ***Significantly different from WT same stage, *P *<* *0.001. Other abbreviations as in Figs 2 and 3.

Myoarchitectural disarray index colour maps and data in fetal mutants (Figs 3 and 4e,f) show that both the number and size of LV areas with myoarchitectural disarray (red/burgundy) exceeded those in wild‐type mice. We also observed a significant difference in the patterns of distribution of disarray, with heterozygous disarray being limited to the anterior, septal and inferior walls, whereas in homozygous mice it involved the whole LV circumference including the lateral wall (Table 1; Figs 3, 4, 6 and 7). Analysis of MDI histograms (Fig. 8; Supporting Information Fig. S6) confirm significant MDI differences between mutants and wild‐type mice irrespective of developmental stage (all *P* < 0.001).

### Myoarchitectural disarray was weakly correlated to wall thickness

At E18.5 we found a small but significant correlation between LV wall thickness and myoarchitectural disarray (lower MDI, i.e. negative correlation) in the inner, mid‐outer and outer layers across all murine groups, but especially in homozygous mice (Table 3). At P0, there was no correlation between MDI and LV wall thickness in wild‐type, but a significant correlation persisted in the inner and mid‐outer layers of heterozygous and homozygous mice (Table 3).

**Table 3:** Correlation (*r*) between wall thickness and MDI^a^

LV Layer	Wild‐type		Heterozygous		Homozygous	
	E18.5	P0	E18.5	P0	E18.5	P0
Outer	−0.15**	0.12	−0.25*	−0.16**	−0.20*	−0.01
Mid‐outer	−0.29*	0.06	−0.16**	−0.22*	−0.29*	−0.11***
Mid‐inner	−0.02	0.06	−0.04	0.03	−0.01	0.11
Inner	0.18**	0.02	0.14***	0.19*	0.23*	0.15**

E, embryonic day; HREM, high‐resolution episcopic microscopy; LV, left ventricle; WT, wall thickness.

^1^Low MDI denotes more myoarchitectural disarray.

*P <* *0.001.

**P <* *0.01.

***P <* *0.05.

## Discussion

In the present study we show for the first time that myoarchitectural disarray is detectable *in utero* in a mouse model of HCM that lacked or expressed reduced levels of MyBP‐C protein in the heart. In heterozygous mice, which mimic cMyBP‐C haploinsufficiency in human HCM patients, myoarchitectural disarray is established before the development of LVH and already noticeable before birth. We provide quantitative evidence that heterozygous and homozygous mice both show loss of the normal, smooth, transmural change in myocyte orientation and a greater burden of disarray compared with wild‐type mice before birth. These findings are notable because they provide insights into the timing of onset of HCM disease with relevance to the human condition. We also show that in wild‐type mice, minute foci of myoarchitectural disarray do occur but are limited to the septal insertion points of the RV.

It has been shown that even before the development of LVH, there exists in humans a detectable subclinical HCM phenotype consisting of crypts, elongation of the anterior mitral valve leaflet, increased LV apical trabecular complexity and smaller LV systolic volume, with the first two being the strongest predictors for carrying sarcomere gene mutations (Captur et al. 2014). More recently, our group reported that in this animal model, there was a measurable embryological HCM phenotype recapitulating at least some of the features observed in patients with subclinical HCM (Captur et al. 2016). In this study, we went further, showing how fetal hearts of our *Mybpc3*‐targeted KOs also exhibit myoarchitectural disarray. The findings are timely as they corroborate recently published strain data in subclinical HCM patients that uncovered abnormalities in myocardial mechanics before the development of LVH (Williams et al. 2018).

Disarray in HCM is considered to arise from a combination of cardiomyocyte and myofibrillar disorientation which, by light microscopy, means that cardiomyocytes and myofibrils lie at angles of up to 90° to each other, as opposed to the normal pattern in which the orientation within a given layer is fairly parallel (Adomian & Beazell, 1986); by electron microscopy misalignment of Z‐discs is seen in adjacent myofibrils. As it is well known that stress vectors influence the orientation of various structural proteins (Adomian & Beazell, 1986), it is plausible that in HCM the orientation of myofibrils in the developing heart could be altered on account of aberrant cardiac contraction geometry.

We know from previous light and electron microscopy work that gross cardiomyocyte and myofibrillar disarray is absent in the 40‐week‐old wild‐type mouse heart (Cannon et al. 2015) but in the healthy human heart, small areas of myocyte disarray are common at the superior and inferior RV insertion points (Kuribayashi & Roberts, 1992; Maron et al. 1992; Hughes, 2004). Not surprisingly, these are also a common sites for focal myocardial fibrosis discovered at the time of late gadolinium enhancement cardiovascular magnetic resonance imaging *in vivo* and commoner still in some forms of congenital (Bulkley et al. 1983) and acquired heart disease (Van Der Bel‐Kahn, 1977; St John Sutton et al. 1980). Unlike in HCM, these small foci of insertion point disarray occurring in otherwise healthy hearts, lack the other histopathological features of HCM such as myocyte hypertrophy and mitochondrial abnormalities. Other regions that are also known to show spotty cardiomyocyte disarray in the normal heart include the trabeculations and areas adjacent to blood vessels, both of which have been seen on histological analysis (Kuribayashi & Roberts, 1992; Hughes, 2004) and corroborated by our MDI data in wild‐type mice.

Our study provides further evidence on how the eigen‐decomposition of the 3D structure tensor applied to HREM images can reveal information regarding the arrangement of the cardiomyocytes in all three directions, measuring their helical and transmural orientations. As stated in the recent work of Stephenson et al. (2018), with the new high‐resolution imaging techniques such as X‐ray micro‐computed tomography, X‐ray phase contrast synchrotron imaging or HREM, now it is possible to resolve the controversy regarding the transmural angulation of the cardiomyocytes. Our results agree with previous studies confirming the existence of a high proportion of cardiomyocytes deviating markedly from the epicardial tangential plane. Specifically, in our wild‐type mouse, on average, 50% of the myocytes exhibited an angle of intrusion or extrusion of up to ± 15**°**, 44.5% between ± 15**°** and ± 45**°**, and 5.5% exceeded an angle of ±45**°**. These values are very similar to those reported in previous studies based on the sectioning of ventricular tissue blocks using circular knives and DT‐MRI (Lunkenheimer et al. 2006; Schmid et al. 2007; Stephenson et al. 2018). We have also shown that the mean angle of intrusion changes from positive values in the base towards negative values in the midventricle and apex in all the study groups, which is consistent with previous studies (Lunkenheimer et al. 2006; Teh et al. 2016). On the other hand, the angle of intrusion was more heterogeneously distributed in the mutant hearts, which agrees with a previous study on a hypertrophic murine model based on the administration of angiotensin II (Schmitt et al. 2009). This angle of intrusion has been suggested to underscore the existence of auxotonic as opposed to unloading ventricular forces (Lunkenheimer et al. 2018; Stephenson et al. 2018). Those cardiomyocytes exhibiting a significant transmural orientation have both constrictive and dilating force components, and the ratio of those components varies locally according to the prevalence of angles of intrusion or extrusion. It has been hypothesised that such mural antagonism serves to stabilise ventricular shape and size, to sustain late systolic dilation, to confine the amplitude of ventricular mural thickening, and to facilitate and enhance the spiralling intracavity flow (Lunkenheimer et al. 2018). Finally, some authors have also described the existence of myocardial ‘sheets’ or ‘sheetlets,’ thus proposing a third angle to describe the orientation of myocytes (McGill et al. 2013; Agger et al. 2017; Nielles‐Vallespin et al. 2017; Stoeck et al. 2018). Although myocytes aggregate to form bunches, we did not find any visual evidence in our HREM images to support myocardial sheets and therefore we have not calculated the ‘sheet’/‘sheetlet’ angle. This could be due to the limited resolution of HREM images to identify myocyte aggregates in the fetal mice heart.

We demonstrate that our reimplementation of the MDI for structure tensors, which measures the similarity alignment of myocytes between adjacent voxels, is a good descriptor of myoarchitectural disarray. This index has been used in the past to characterise brain tissue organisation (Wu et al. 2004; Wang et al. 2008) and more recently to quantify myocardial disarray in hypertensive rat hearts by DT‐MRI (Giannakidis et al. 2012), where it was superior to fractional anisotropy as it provided more explicit information about irregularity. A key novelty of our work is the application of MDI to HREM enabling us to study the fetal and neonatal heart. By MDI, we show that disarray was significantly higher in homozygous than wild‐type or heterozygous mice, not just in the septum, but also in LV lateral wall, with an overall reduction of the amount of circumferentially arranged cardiomyocytes. Results are concordant with previous histological reports of myocyte disorganisation in HCM extending to the anterolateral and posterior LV walls (Maron et al. 1992), and with the loss of the midwall circumferential layer (Kuribayashi & Roberts, 1992). Moreover, our results agree with recent work using generalised Q‐space imaging applied to three homozygous *Mybpc3* mouse models at post‐natal weeks 10–12, which also showed varying degrees of global myoarchitectural disarray (Taylor et al. 2016) with disruption of overall helical patterns and reduced slope of the HA transmural profile in the homozygous *Mybpc3* knock‐out mouse.

Previous studies that investigated the association between LVH and myocardial disorganisation failed to find a significant correlation between the two (maximum *r*: −0.159) (Maron et al. 1992). However, these studies were based on a macroscopic visual qualitative appraisal of histological section images and were missing detailed myoarchitectural disarray quantification. Using HREM and a 45‐segment fully quantitative approach, we found even in wild‐type mice a weak but significant correlation between myoarchitectural disarray and wall thickness in all but the mid‐inner layer (maximum *r* −0.29; minimum *r* −0.11). This supports the notion that myoarchitectural disarray is not confined to greatly thickened sections of the ventricular myocardium, but that regions of relatively normal or borderline increased wall thickness can also show measurable myoarchitectural disarray (Maron et al. 1992).

Limitations of the study include not performing deep phenotyping of mutant mice earlier than E18.5. Some other histological aspects of the extended HCM phenotype (Olivotto et al. 2009) such as interstitial fibrosis and coronary arteriolar changes have not been reported in this work. We might expect differences between *in vivo* and *ex vivo* data, and also between fresh and fixed *ex vivo* data. Although there are studies that have described that formalin‐fixation affects diffusion properties and affect the microstructural organisation of cardiac tissue, there are no studies investigating the effects of fixation or dehydration on the structure tensor (Giannakidis et al. 2016; Mazumder et al. 2016). As stated by Giannakidis et al. (2016) it is crucial to minimise the time of the start of tissue fixation after death to ensure that *ex vivo* measurements represent the structural characteristics of the living myocardium as accurately as possible. Further research is warranted to investigate how the different preservation procedures such as fixation or freezing affect measurements of cardiomyocyte orientation based on structure tensor methods. However, as wild‐type and mutant hearts were treated in the exactly same way, we are confident about inter‐group differences. Another limitation of the present study is that we have not taken into account the epicardial curvature when quantifying the helical and transmural angulation of the myocytes, which may lead to errors in the measurement of both angles in the areas of high curvature such as the apex or the base. However, although the absolute values of the angles may not be fully correct in the more apical and basal slices, as wild‐type and mutant hearts were analysed in the exact same way, the relative differences between groups described in this study will remain the same.

In assessing translational relevance, we must bear in mind that mice and humans differ, yet we have chosen a faithful model of human HCM: we know these heterozygous mice go on in later life to exhibit asymmetric septal hypertrophy (Carrier et al. 2004) akin to the usual adult human HCM (Ho et al. 2009; Michels et al. 2009), whereas homozygous mice develop LVH, systolic dysfunction and histopathological features of HCM from 3 weeks after birth. Future work should be directed at elucidating the mechanism/s by which cMyBP‐C protein haploinsufficiency in this model is altering the cardiac developmental trajectory, leading to fetal disarray, extending investigations to other HCM mouse models with other mutations, and understanding the impact of mouse genetic background on phenotype expression.

In conclusion, our animal data provide the first quantitative evidence that myoarchitectural disarray in HCM begins *in utero* and can be measured from HREM data. Findings should stimulate further research in this area with the potential to improve our understanding of the pathogenesis and molecular mechanisms of HCM.

## Conflict of interest

The authors declare no conflict of interest.

## Authors’ contributions

P.G.C. and A.C.C. developed the concept and approach; P.G.C. performed the statistical analysis; P.G.C. and G.C. wrote the paper; G.C. performed the murine harvesting in Hamburg and heart preparation for HREM; T.J.M. led the HREM dissections in the UK; J.C.M., A.C., L.C., T.J.M., O.O. and W.J.M. provided expert review of the manuscript; L.C. and S.S. provided the heterozygous and homozygous KO HCM mutant and wild‐type embryos; all authors reviewed and approved the final manuscript.

## Supporting information


**Video S1.** Fibre tracking of wild‐type (WT) mouse heart at embryonic (E) day 18.5 based on ***v***_3_ eigenvector. Tracks were colour‐coded by z‐component value of the unit ***v***_3_.

**Video S2.** Fibre tracking of heterozygous (HET) knock‐out mouse heart at embryonic (E) day 18.5 based on ***v***_3_ eigenvector. Tracks were colour‐coded by z‐component value of the unit ***v***_3_.

**Video S3.** Fibre tracking of homozygous (HO) knock‐out mouse heart at embryonic (E) day 18.5 based on ***v***_3_ eigenvector. Tracks were colour‐coded by z‐component value of the unit ***v***_3_.

**Appendix S1.** Calculation of the cardiomyocyte's orientation.
**Appendix S2.** Calculation of the myocardial disarray index.
**Table S1.** 45‐segment HREM wall thickness data in wild‐type, heterozygous and homozygous mice.
**Fig. S1.** Definition of the 45‐segment approach.
**Fig. S2**. Segment‐specific transmural profiles of helical angle (HA) in the LV.
**Fig. S3**. Segment‐specific transmural profiles of intrusion angle (IA) in the LV.
**Fig. S4.** Segment‐specific distributions of helical angle (HA) in the LV.
**Fig. S5.** Segment‐specific distributions of intrusion angle (IA) in the LV.
**Fig. S6.** Segment‐specific distributions of myoarchitectural disarray index (MDI) in the LV.
