# Supplementary material for: Myoarchitectural disarray of hypertrophic cardiomyopathy begins pre‐birth
Source: J Anat. 2019 Jul 26;235(5):962–76. doi: 10.1111/joa.13058 (PMC6794206; doi:10.1111/joa.13058)
Supplement: Supplementary file 4 — Appendix S1. Calculation of the cardiomyocyte's orientation. Appendix S2. Calculation of the myocardial disarray index. Table S1. 45‐segment HREM wall thickness data in wild‐type, heterozygous and homozygous mice. Fig. S1. Definition of the 45‐segment approach. Fig. S2. Segment‐specific transmural profiles of helical angle (HA) in the LV. Fig. S3. Segment‐specific transmural profiles of intrusion angle (IA) in the LV. Fig. S4. Segment‐specific distributions of helical angle (HA) in the LV. Fig. S5. Segment‐specific distributions of intrusion angle (IA) in the LV. Fig. S6. Segment‐specific distributions of myoarchitectural disarray index (MDI) in the LV. [file JOA-235-962-s004.docx]

SUPPLEMENTARY MATERIAL

**Myoarchitectural disarray of hypertrophic cardiomyopathy begins pre-birth­**

Patricia Garcia Canadilla* PhD,^1^ Andrew C Cook* PhD,^1^ Timothy J Mohun PhD,^2^ Onyedikachi Oji MBBS MSc,^1^ Saskia Schlossarek PhD,^3,4^ Lucie Carrier PhD,^3,4^ William J McKenna BA MD DSc FRCP FMedSci FESC FACC,^1^ James C Moon** MD, MBBS, MRCP,^1,5^ Gabriella Captur** MD PhD MRCP MSc.^1^

*Joint first authors

**Joint last authors

1. Institute of Cardiovascular Science, University College London, Gower Street, London, UK
2. The Francis Crick Institute, Midland Place, London, UK
3. Institute of Experimental Pharmacology and Toxicology, Cardiovascular Research Center, University Medical Center Hamburg-Eppendorf, Hamburg, Germany
4. DZHK (German Center for Cardiovascular Research), partner site Hamburg/Kiel/Lübeck, Hamburg, Germany
5. Barts Heart Center, The Cardiovascular Magnetic Resonance Imaging Unit, St Bartholomew’s Hospital, West Smithfield, London, UK

**Emails:** p.canadilla@ucl.ac.uk; a.cook@ucl.ac.uk; tim.mohun@crick.ac.uk; onyedikachi.oji.16@ucl.ac.uk; sschlossarek@uke.de; l.carrier@uke.de; w.mckenna@ucl.ac.uk; j.moon@ucl.ac.uk

**SHORT TITLE: HCM disarray in the fetal heart**

**Supplementary Methods**

1. **Calculation of the cardiomyocyte’s orientation**

Briefly, for each image voxel, the gradient in *x*, *y* and *z* directions was obtained using a central difference algorithm. The structure tensor (T) was calculated as the cross product of gradient vectors as follows:

Eigen-decomposition was then applied to the structure tensor T, to obtain the three eigenvalues and eigenvectors. The eigenvector (*v_3_*) with the smallest eigenvalue (*λ_3_*) (tertiary eigenvector) was considered to point in the direction of the long axis of the myocytes as it corresponds with the direction with lowest image intensity variation (Figure 1a).

We then calculate the helical (HA) and intrusion angle (IA) of the cardiomyocytes in each voxel. To do that, we performed first a change of the coordinates system from cartesian to prolate spheroidal coordinates (**Figure 1b**). The prolate spheroidal coordinates system have the advantage of been physiologically meaningful with respect to the LV ellipsoidal shape (Toussaint et al., 2013). To do that, we calculated the semi-foci distance *f* for each dataset as follows:

$$f=\sqrt{R_{a}^{2}-R_{b}^{2}}$$

where *R_a_* and *R_b_* are the major and minor axis of the ellipsoid respectively.

1. **Calculation of the myocardial disarray index**

MDI is an anisotropy measure of the angular uniformity of myocytes in a neighbourhood. We considered a neighbourhood of 49.3 x 49.3 x 49.3 µm^3^; this was large enough to cover ~48 cardiomyocytes (cf. the volume of a fetal mouse cardiomyocyte is ~2500 µm^3^) (Bensley et al., 2016) but small enough to resolve local information on myocytes organisation. In contrast to DT-MRI studies where disarray is described by the scatter matrix considering the primary diffusion eigenvector (Giannakidis et al., 2012; Wang et al., 2008; Wu et al., 2004), we used the tertiary eigenvector (*v_3_*) of the structure tensor to compute the scatter matrix. Therefore, the scatter matrix (S) was computed as:

$$S=\frac{1}{n}\sum_{i=1}^{n} v_{3i}v_{3i}^{'}$$

where each element of the scatter matrix S contains products of the *x*, *y* and *z* components of the tertiary eigenvector *v_3i_*. An eigen-decomposition of the scatter matrix was then performed and the three eigenvalues (*t*_i_) obtained. MDI was thus computed as:

whereis the mean eigenvalue of the scatter matrix *S*. MDI values range from 0 (complete anisotropy) to 1 (isotropy). A large MDI indicates a voxel with highly coherent or colinear eigenvector organisation and therefore, insignificant disarray. Voxels with low MDI have a noncollinear distribution of eigenvectors indicating loss of myocardial organisation and therefore disarray.

**Supplementary Tables**

**Table S1. 45-Segment HREM wall thickness data in wildtype, heterozygous and homozygous mice.**

|  | | **Wildtype** | | **Heterozygous** | | **Homozygous** | |  |
| --- | --- | --- | --- | --- | --- | --- | --- | --- |
|  |  | **E18.5** | **P0** | **E18.5** | **P0** | **E18.5** | **P0** |  |
| Nº of mice | | 8 | 4 | 7 | 9 | 18 | 10 |  |
| LV mid AP lumen (mm) | | 0.96 ± 0.10 | 1.01 ± 0.15 | 1.02 ± 0.04 | 0.91 ± 0.08 | 0.93 ± 0.07 | 0.86 ± 0.11‡ |  |
| *LV max. septal WT^∞^ (mm)* | | | | | | | | |
|  | Level-1 | 0.495 ± 0.059 | 0.598 ± 0.133 | 0.507 ± 0.077 | 0.568 ± 0.040 | 0.528 ± 0.044 | 0.566 ± 0.053 |  |
|  | Level-2 | 0.481 ± 0.022 | 0.603 ± 0.102 | 0.527 ± 0.079 | 0.661 ± 0.039 | 0.570 ± 0.046‡ | 0.580 ± 0.068~ |  |
|  | Level-3 | 0.566 ± 0.033 | 0.645 ± 0.051 | 0.577 ± 0.061 | 0.659 ± 0.067 | 0.573 ± 0.042 | 0.582 ± 0.075~ |  |
|  | Level-4 | 0.520 ± 0.065 | 0.648 ± 0.135 | 0.527 ± 0.077 | 0.602 ± 0.039 | 0.587 ± 0.049 | 0.565 ± 0.066 |  |
|  | Level-5 | 0.458 ± 0.026 | 0.585 ± 0.071 | 0.444 ± 0.061 | 0.577 ± 0.044 | 0.540 ± 0.064\|\| | 0.532 ± 0.072 |  |
| *LV max. lateral WT^≠^ (mm)* | | | | | | | | |
|  | Level-1 | 0.339 ± 0.059 | 0.443 ± 0.099 | 0.401 ± 0.55 | 0.416 ± 0.054 | 0.415 ± 0.068 | 0.465 ± 0.068 |  |
|  | Level-2 | 0.369 ± 0.033 | 0.470 ± 0.123 | 0.344 ± 0.039 | 0.434 ± 0.036 | 0.440 ± 0.074 | 0.423 ± 0.063 |  |
|  | Level-3 | 0.378 ± 0.025 | 0.493 ± 0.079 | 0.351 ± 0.035 | 0.431 ± 0.036 | 0.402 ± 0.040 | 0.429 ± 0.062 |  |
|  | Level-4 | 0.379 ± 0.021 | 0.525 ± 0.037 | 0.350 ± 0.051 | 0.419 ± 0.036 | 0.424 ± 0.040 | 0.440 ± 0.063 |  |
|  | Level-5 | 0.428 ± 0.038 | 0.563 ± 0.104 | 0.387 ± 0.062 | 0.470 ± 0.041 | 0.435 ± 0.038 | 0.459 ± 0.064 |  |

E = embryonic day; LV = left ventricle; AP = anteroposterior; WT = wall thickness. All values are expressed as mean ± standard deviation unless otherwise stated.

∞Segments 1, 6, 7, 8 and 9 are considered in the septal group.

≠Segments 2, 3, 4 and 5 are considered in the lateral group.

*Significantly different from Wildtype same stage, *p* < 0.001.

†Significantly different from Wildtype same stage, *p* < 0.01.

‡Significantly different from Wildtype same stage, *p* < 0.05.

§Significantly different from HET same stage, *p* < 0.001.

||Significantly different from HET same stage, *p* < 0.01.

~Significantly different from HET same stage, *p* < 0.05.

**Supplementary Figures:**

**Figure S1. Definition of the 45-segment approach**. To ensure higher resolution and more complete left ventricle (LV) coverage for the myoarchitectural disarray index (MDI) measurements, we considered 5 LV slices from base to apex, each with 9 segments, generating a total of 45 myocardial segments. Each segment was further subdivided into 4 layers: inner, mid-inner, mid-outer and outer.

**
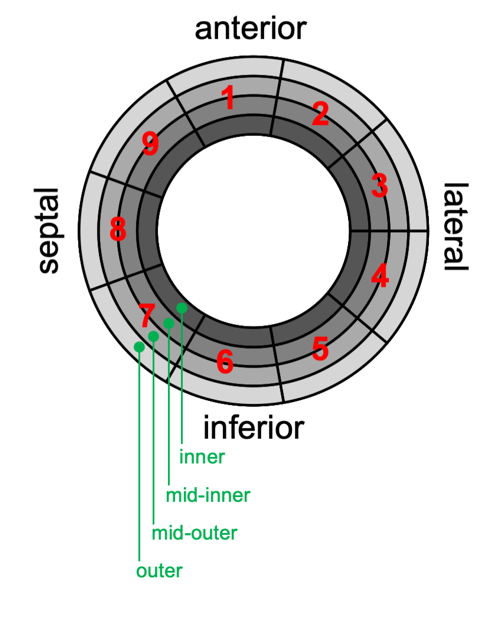
**

**Figure S2**. **Segment-specific transmural profiles of helical angle (HA) in the LV.** Left ventricular (LV) transmural profiles of helical angle (HA) as a function of normalised myocardial depth from endocardium (0) to epicardium or right-ventricular endocardium in the septum (1) in all the 16 LV segments (considering the 17-segment model, excluding the apical cap [segment 17]) in wildtype (WT), heterozygous (HET) and homozygous (HO) knock-out mouse hearts at embryonic day (E) 18.5 (**a**) and post-natal day (P) 0 (**b**). Solid lines: group means. ﻿Ribbons: +/- standard deviation.

**
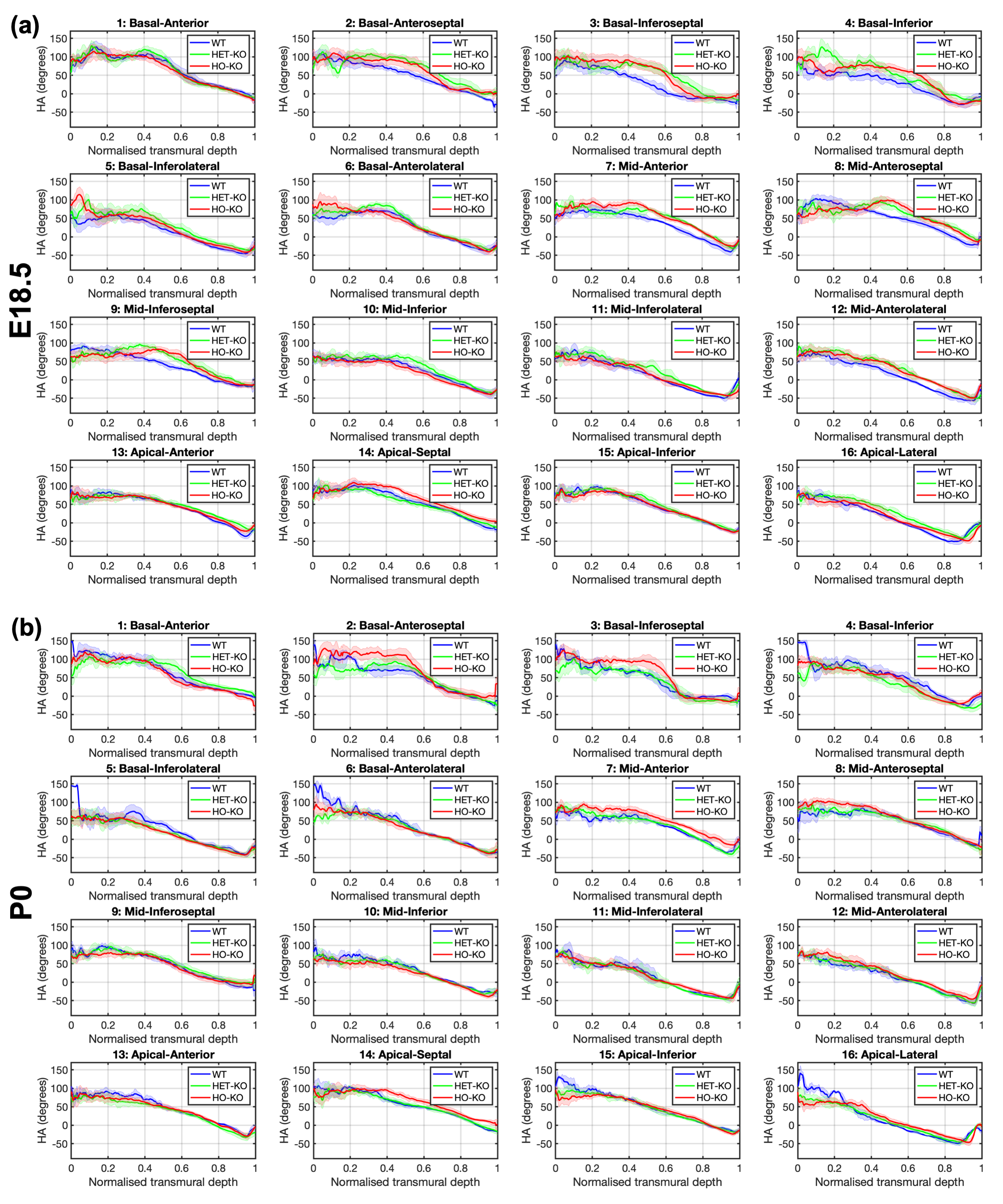
**

**Figure S3**. **Segment-specific transmural profiles of intrusion angle (IA) in the LV.** Left ventricular (LV) transmural profiles of intrusion angle (IA) as a function of normalised myocardial depth from endocardium (0) to epicardium or right-ventricular endocardium in the septum (1) in all the 16 LV segments (considering the 17-segment model, excluding the apical cap [segment 17]) in WT, HET and HO knock-out mouse hearts at E18.5 (**a**) and P0 (**b**). Solid lines: group means. ﻿Ribbons: +/- standard deviation. Other abbreviations as in **Figure S2**.

**
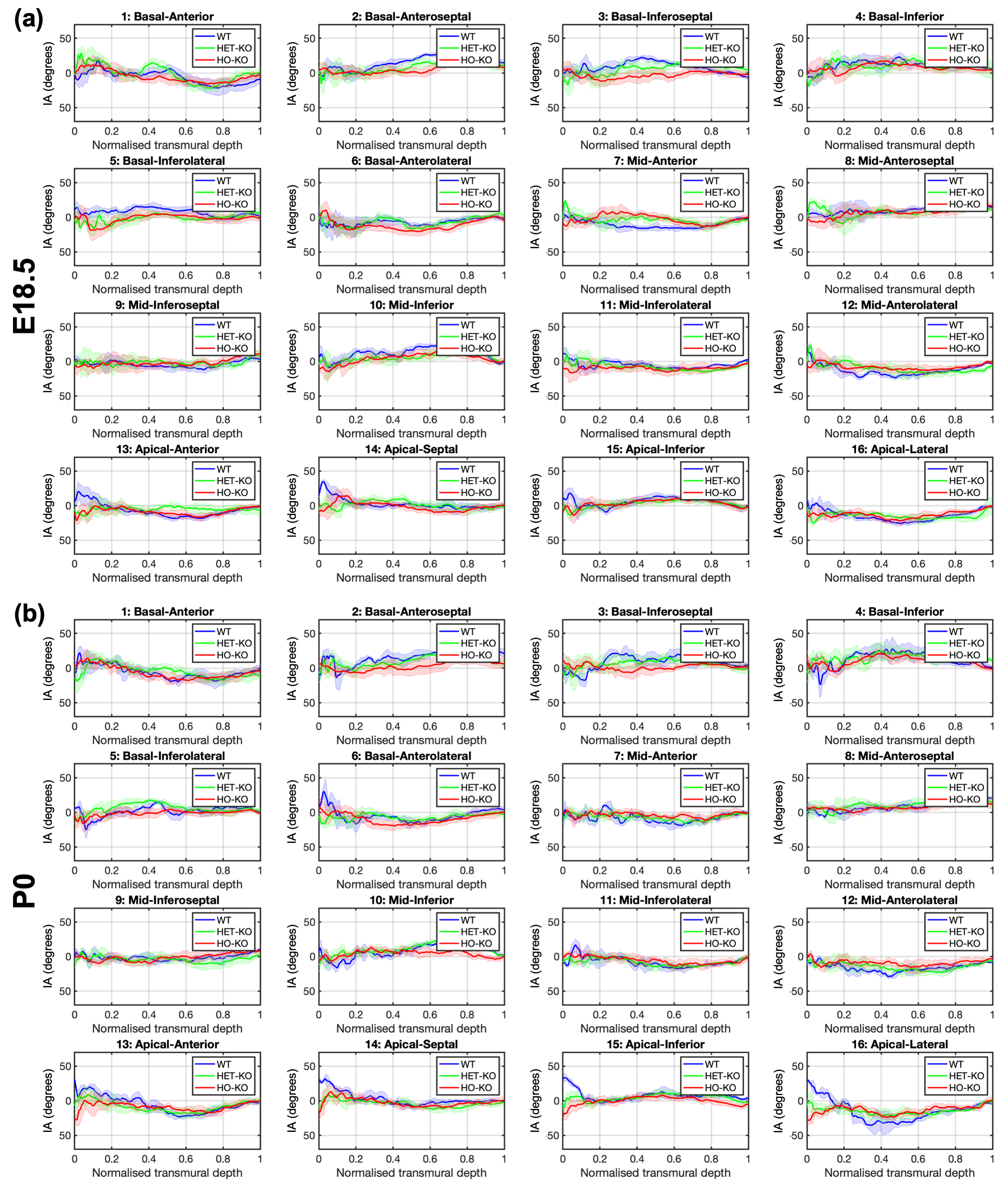
**

**Figure S4.** **Segment-specific distributions of helical angle (HA) in the LV.** Left ventricular (LV) distributions of HA across the 16 LV segments (considering the 17-segment model, excluding the apical cap [segment 17]) in WT, HET and HO knock-out mouse hearts at E18.5 (**a**) and P0 (**b**). *Significantly different from WT same stage, *p* < 0.05; **Significantly different from WT same stage, *p* < 0.01; ***Significantly different from WT same stage, *p* < 0.001. Abbreviations as in **Supplementary Figures S1 and S2**.

**
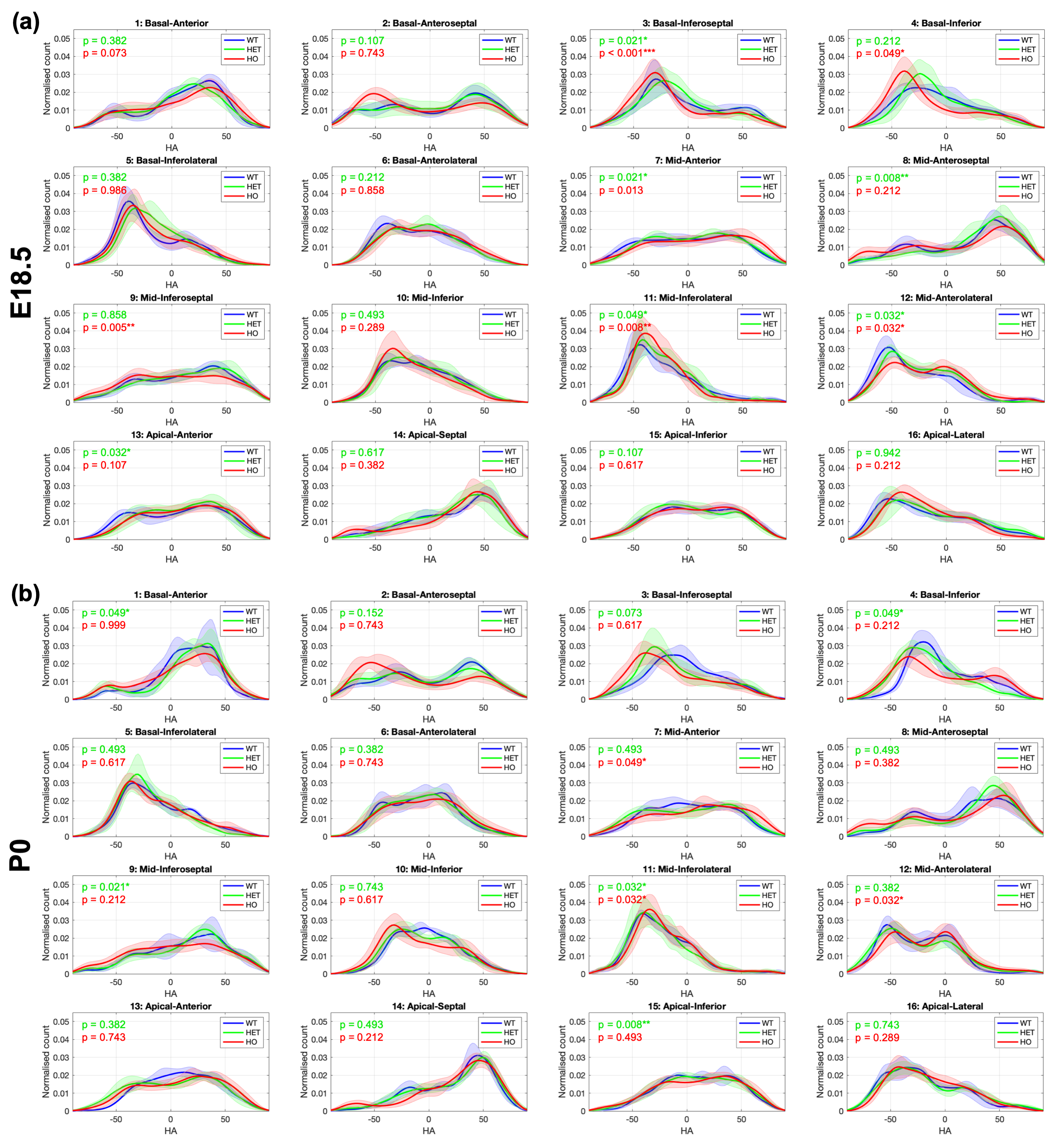
**

**Figure S5.** **Segment-specific distributions of intrusion angle (IA) in the LV.** Left ventricular (LV) distributions of intrusion angle (IA) across the 16 LV segments (considering the 17-segment model, excluding the apical cap [segment 17]) in WT, HET and HO knock-out mouse hearts at E18.5 (**a**) and P0 (**b**). *Significantly different from WT same stage, *p* < 0.05; **Significantly different from WT same stage, *p* < 0.01; ***Significantly different from WT same stage, *p* < 0.001. Abbreviations as in **Supplementary Figures S1 and S2**.

**
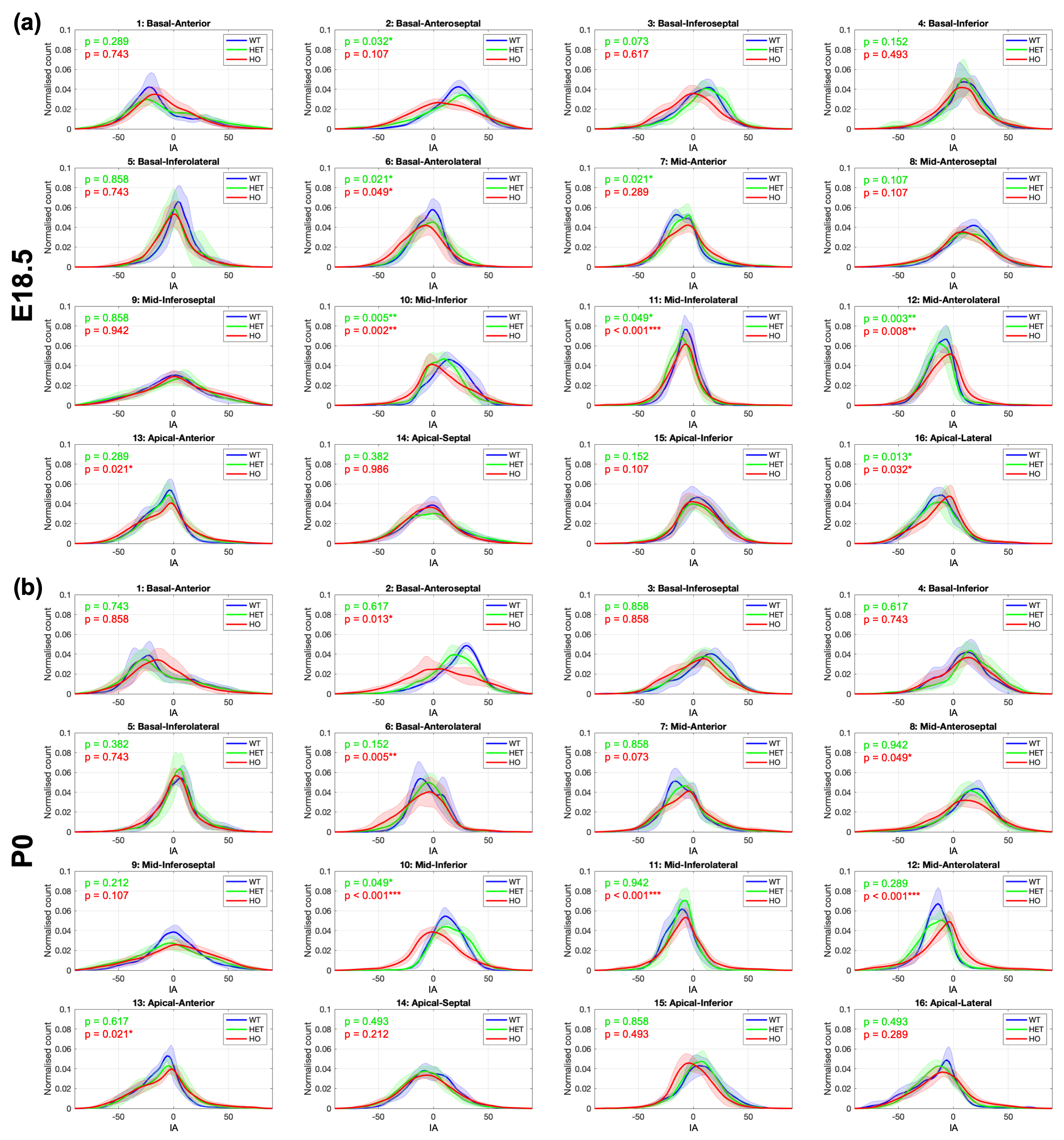
**

**Figure S6.** **Segment-specific distributions of myoarchitectural disarray index (MDI) in the LV.** Left ventricular (LV) distributions of MDI across the 16 LV segments (considering the 17-segment model, excluding the apical cap [segment 17]) in WT, HET and HO knock-out mouse hearts at E18.5 (**a**) and P0 (**b**). *Significantly different from WT same stage, *p* < 0.05; **Significantly different from WT same stage, *p* < 0.01; ***Significantly different from WT same stage, *p* < 0.001. Abbreviations as in **Supplementary Figures S1 and S2**.

**
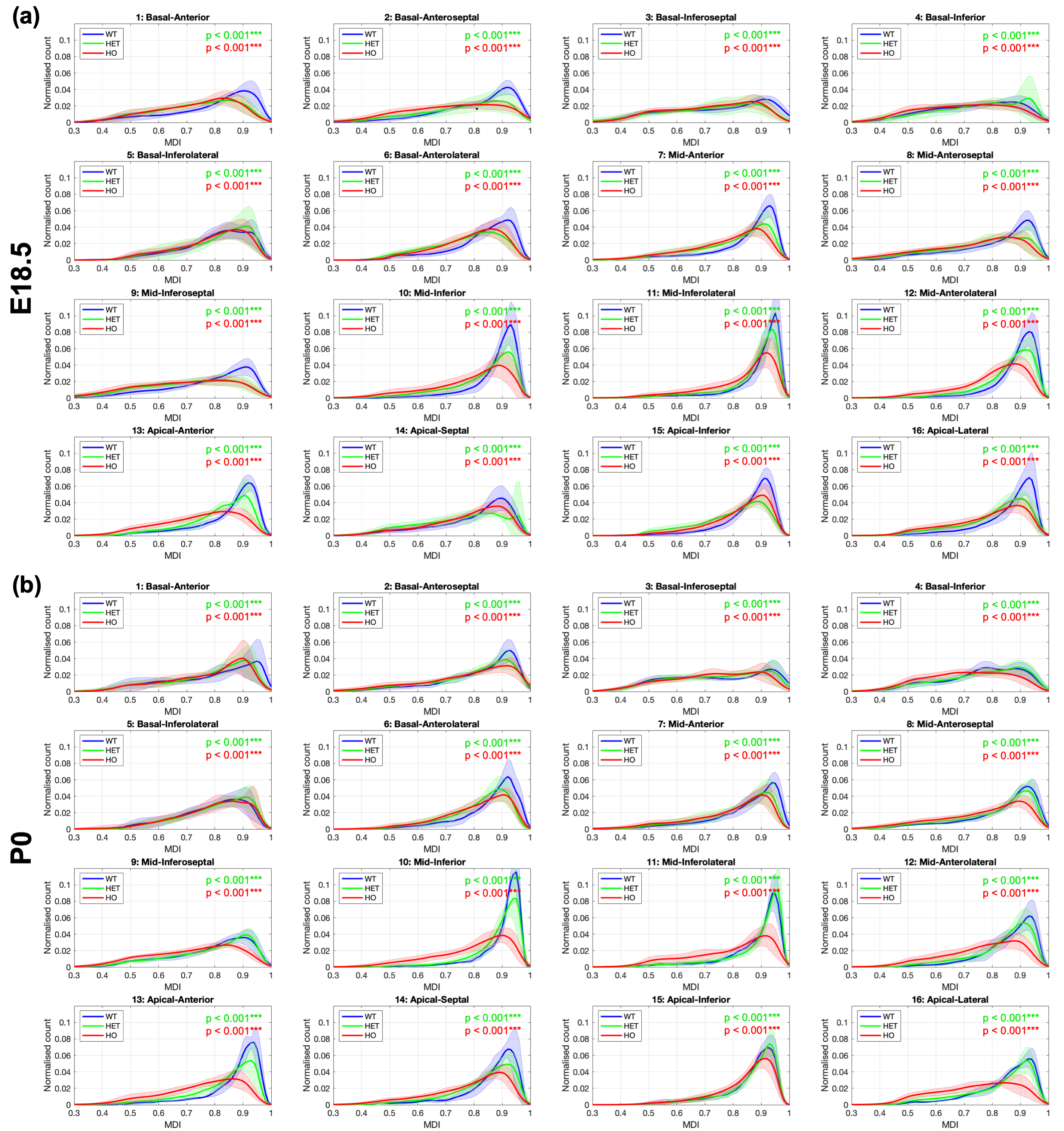
**

**Supplementary Videos:**

**Supplementary Video 1.** Fibre tracking of wildtype (WT) mouse heart at embryonic (E) day 18.5 based on v3 eigenvector. Tracks were colour-coded by z-component value of the unit ***v_3_***.

**Supplementary Video 2.** Fibre tracking of heterozygous (HET) knock-out mouse heart at embryonic (E) day 18.5 based on v3 eigenvector. Tracks were colour-coded by z-component value of the unit ***v_3_***.

**Supplementary Video 3.** Fibre tracking of homozygous (HO) knock-out mouse heart at embryonic (E) day 18.5 based on v3 eigenvector. Tracks were colour-coded by z-component value of the unit ***v_3_***.

**REFERENCES**

Bensley, J.G., De Matteo, R., Harding, R., Black, M.J., 2016. Three-dimensional direct measurement of cardiomyocyte volume, nuclearity, and ploidy in thick histological sections. Sci. Rep. 6, 23756.

Giannakidis, A., Rohmer, D., Veress, A.I., Gullberg, G.T., 2012. Diffusion Tensor Magnetic Resonance Imaging-Derived Myocardial Fiber Disarray in Hypertensive Left Ventricular Hypertrophy: Visualization, Quantification and the Effect on Mechanical Function. In: Shenasa, M., Hindricks, G., Borggrefe, M., et al. (Eds.), Cardiac Mapping. Wiley, pp. 574–588.

Toussaint, N., Stoeck, C.T., Schaeffter, T., et al., 2013. In vivo human cardiac fibre architecture estimation using shape-based diffusion tensor processing. Med. Image Anal. 17, 1243–1255.

Wang, J., Lin, Y., Wai, Y., et al., 2008. Visualization of the coherence of the principal diffusion orientation: An eigenvector-based approach. Magn. Reson. Med. 59, 764–770.

Wu, Y.-C., Field, A.S., Chung, M.K., Badie, B., Alexander, A.L., 2004. Quantitative analysis of diffusion tensor orientation: theoretical framework. Magn Reson Med 52, 1146–1155.
